# Supplementary material for: The miR-27a/FOXJ3 Axis Dysregulates Mitochondrial Homeostasis in Colorectal Cancer Cells
Source: Cancers (Basel). 2021 Oct 5;13(19):4994. doi: 10.3390/cancers13194994 (PMC8507763; doi:10.3390/cancers13194994)

The miR-27a/FOXJ3 axis dysregulates mitochondrial homeostasis in colorectal cancer cells

Giovannina Barisciano1*, Manuela Leo1*, Livio Muccillo1, Erica Pranzini2, Matteo Parri2, Vittorio Colantuoni1, Maria Letizia Taddei3 and Lina Sabatino1§

Content of Supplementary Information

Supplementary Figure S1

Supplementary Figure S2

Supplementary Table S1

Supplementary Figure S1

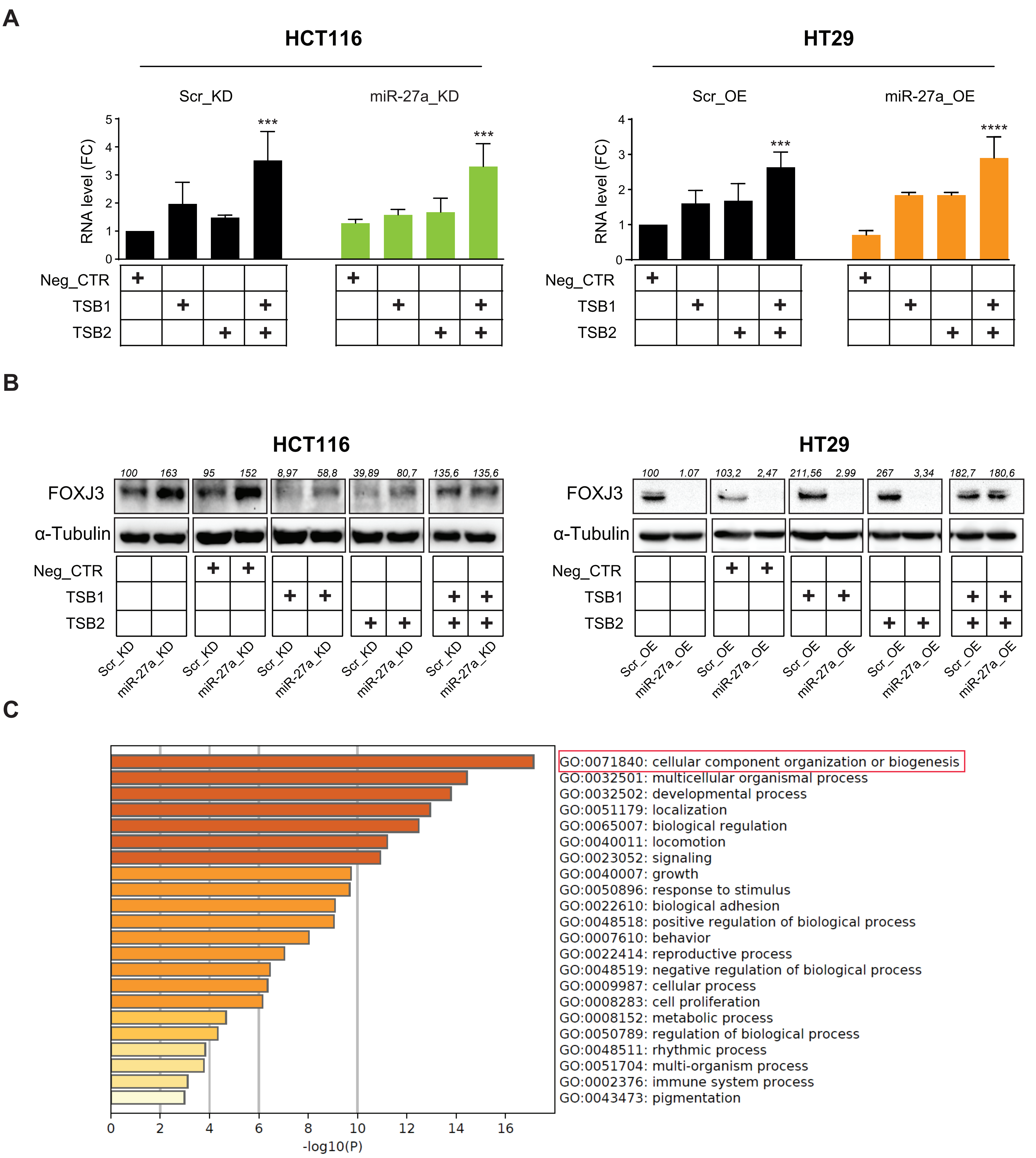

Supplementary Figure S1

Analysis of FOXJ3 expression in the cell model system upon transfection of the Target Site Blockers (TSB).

(A) qRT-PCR analysis of RNA from Scr_KD or miR-27a_KD and Scr_OE or miR-27a_OE cells transfected with TSB1 and TSB2, directed against the seed-sequences recognized by miR-27a on FOXJ3 mRNA, or the negative control.

(B) Western blot analysis for FOXJ3 on extracts from Scr_KD or miR-27a_KD and in Scr_OE or miR-27a_OE cells transfected as in A.

(C) Gene ontology analysis of the major pathways modulated by miR-27a according to the TCGA-COADREAD dataset patients. The graph bars illustrate the most influenced pathways among which “cellular component organization or biogenesis” is the top one (a deeper colour indicates a smaller p-value).

Supplementary Figure S2

miRNA: hsa-miR-27a_3p ***Sequence:*** *3' CGCCUUGAAUCGGUGACACUU 5'*

Predicted target		*Sequence*	*Energy*
PGC1-α NM_001330751.2	PGC1-α:<br>hsa-miR-27a_3p	TGAGTGAATCCACTGTTGTC<br>CGCCUUGAAUCGGUGACACUU	Energy-7.26 kcal/mol<br>Hybridization Energy-10.31 kcal/mol<br>Unfolding Energy - Target1.09 kcal/mol<br>Unfolding Energy - Query1.96 kcal/mol
	PGC1-α:<br>hsa-miR-27a_3p	ATGAACACAGCTGCTGAAGAG<br>CGCCUUGAAUCGGUGACACUU	Energy-9.58 kcal/mol<br>Hybridization Energy-14.59 kcal/mol<br>Unfolding Energy - Target3.04 kcal/mol<br>Unfolding Energy - Query1.97 kcal/mol
	PGC1-α:<br>hsa-miR-27a_3p	AGTCAAGCCACTACAGA<br>CGCCUUGAAUCGGUGACACUU	Energy-9.2 kcal/mol<br>Hybridization Energy-11.86 kcal/mol<br>Unfolding Energy - Target0.7 kcal/mol<br>Unfolding Energy - Query1.96 kcal/mol
	PGC1-α:<br>hsa-miR-27a_3p	AGAGTAACAGCCGCTGGCGA<br>CGCCUUGAAUCGGUGACACUU	Energy-8.6 kcal/mol<br>Hybridization Energy-13.87 kcal/mol<br>Unfolding Energy - Target3.3 kcal/mol<br>Unfolding Energy - Query1.97 kcal/mol
NRF1 NM_001040110.2	NRF1:<br>hsa-miR-27a_3p	ATTTTTTATCGTCATTGTGAA<br>GCCUUGAAUCGGUGACACUU	Energy-9.96 kcal/mol<br>Hybridization Energy-15.09 kcal/mol<br>Unfolding Energy - Target3.16 kcal/mol<br>Unfolding Energy - Query1.97 kcal/mol
	NRF1:<br>hsa-miR-27a_3p	CGGAATTCCAGTCTCTGTGGA<br>CGCCUUGAA UCGGUGACACUU	Energy-10.88 kcal/mol<br>Hybridization Energy-21.69 kcal/mol<br>Unfolding Energy - Target8.84 kcal/mol<br>Unfolding Energy - Query1.97 kcal/mol
TFAM NM_001270782.2	TFAM:<br>hsa-miR-27a_3p	TCCACTCACAGCCATTCTCCCTC<br>CGCCUUGAAUCGGUGACACUU	Energy-7.05 kcal/mol<br>Hybridization Energy-9.6 kcal/mol<br>Unfolding Energy - Target0.58 kcal/mol<br>Unfolding Energy - Query1.97 kcal/mol
	TFAM:<br>hsa-miR-27a_3p	CAGGACAGTGTTGGCCATTGGTGAA<br>CGCCUUG AAUCGGUGAC ACUU	Energy-7.05 kcal/mol<br>Hybridization Energy-9.6 kcal/mol<br>Unfolding Energy - Target0.58 kcal/mol<br>Unfolding Energy - Query1.97 kcal/mol
	TFAM<br>hsa-miR-27a_3p	GCAGAGCTGTGCACCGGCTGTGGA<br>CGCCUUGAAUCGGU GACACUU	Energy-4.53 kcal/mol<br>Hybridization Energy-16.23 kcal/mol<br>Unfolding Energy - Target9.73 kcal/mol<br>Unfolding Energy - Query1.97 kcal/mol

IntaRNA - RNA-RNA interaction http://rna.informatik.uni-freiburg.de/IntaRNA/Input.jsp

Supplementary Figure S2

The putative recognition sequences for miR-27a on the mRNAs corresponding to some components of the mitochondrial biogenetic process are illustrated along with their hybridization free energies.

Supplementary Table S1

Supplementary Table S1. A

miRCURY LNA miRNA Power Target Site Blockers	(Qiagen cod. 339194)
Seq_ Negative Control	ACGTCTATACGCCCA
Seq_TSB1 (794_816)	TCACAGTG TTGATT GCCA GAGTT
Seq TSB2 (3247_3266)	AGGCG CAGTGGCT CATGTCT

Supplementary Table S1. B

Cell Signaling Technology (Beverly MA USA)	Sigma Aldrich (St Louis MO USA)	Bioss antibody (Woburn MA USA)	BETHYL Laboratories (Montgomery TX USA)	ABCAM (Cambridge UK)	Invitrogen ™ (Carlsbad, CA, USA)	Santa Cruz biotechnology (Dallas TX USA)
Mitochondrial Dynamics Antibody Sampler KitII (#74792)	β-Actin (#F-3022)	NRF 1 (#BS-1342R)	TFAM (#A303-226A-T)	OXPHOS WB antibody cocktail (#ab110411)	Alexa Fluor™ 633 (A20005)	PGC1-α (#518025)
Mitophagy Antibody Sampler Kit (#43110)	α-Tubulin (#T5168)		FOXJ3 (#A303-107A-T)			
TOM 20 (#42406)	FOXJ3 (#SAB4500907)					
	TOM 20 (#HPA 011562)					

Supplementary Table S1. C

Gene	Forward Primer	Reverse Primer
FOXJ3	5′ – AGC CTA ACG TCT ATG GAC TGG T – 3′	5′- GGT CAA GGA GTG CAT TCT TCT TA - 3′
PGC1α	5′ – AAG GTC TCC AGG CAG TAG AT-3′	5′ - AAG GGA GAA TTT CGG TGC GT - 3′
NRF1	5′ – TCT CAC CCT CCA AAC CTA AC – 3′	5′ – CCC GAC CTG TAG AGT ACT TG – 3′
TFAM	5′ – GGC AAG TTG TCC AAA GAA ACC – 3′	5′ – GCA TCT GGG TTC TGA GCT TTA - 3′
18S	5′ – GGG AGC CTG AGA AAC GGC -3′	5′ –GGG TCG GGA GTG GGT AAT TT – 3′
SOX9	5′ – CTC AAC GGG TCC CTG AGT TG – 3′	5′ – TCC GTT TTG GTT TTG GAG CG – 3′
Claudin1	5′ – GAT AAT TGG AGT GAA TGA ATG AAA AG – 3′	5′ – GTT TCA GGG CGG CTC AC – 3′
tRNA-Leu	5′ - CAC CCA AGA ACA GGG TTT GT – 3′	TGGCC ATGG ATATGT TGT TA – 3′
MT - RNR 2	5′ - CGA AAG GAC AAG AGA AAT AAG G – 3′	5′ - CTG TAA AGT TTT AAG TTT TAT GCG – 3′

Supplementary Table S1

The table reports the sequences of the Target Site Blockers (TSB) and negative control (1A), the antibodies used throughout the manuscript (1B) and the sequences of the primers used for qRT-PCR and q-PCR (1C).

Original images for blots/gels

The miR-27a/FOXJ3 axis dysregulates mitochondrial homeostasis in colorectal cancer cells

Giovannina Barisciano[1*], Manuela Leo[1*], Livio Muccillo[1], Erica Pranzini[2], Matteo Parri[2], Vittorio Colantuoni[1], Maria Letizia Taddei[3] and Lina Sabatino[1§]

Figure S1C

HCT116 - Scr_KD and miR-27a_KD

FOXJ3

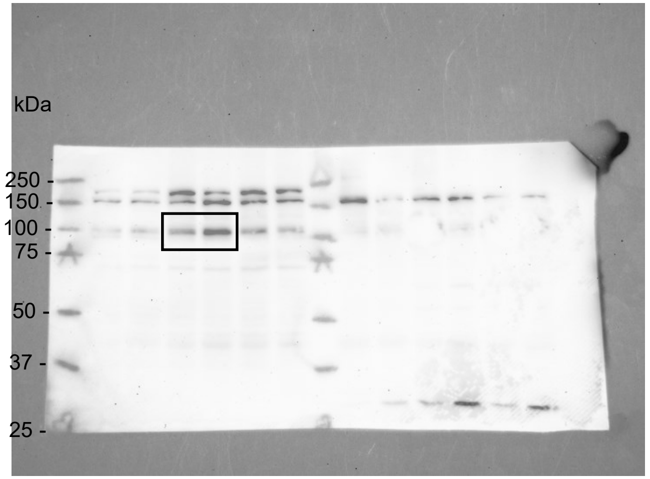

α-Tubulin for FOXJ3

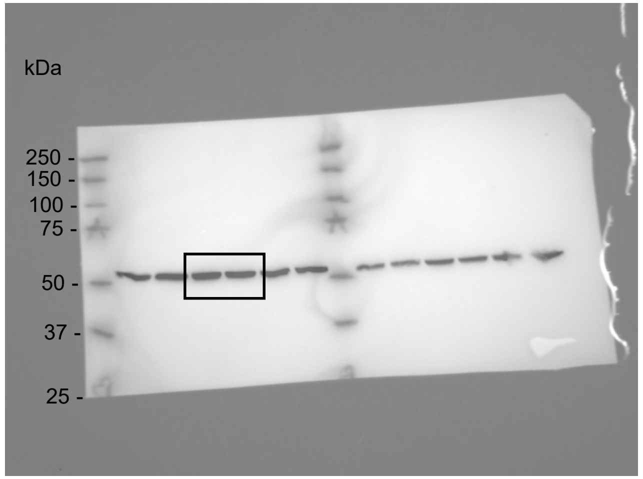

HT29 - Scr_OE and miR-27a_OE

FOXJ3

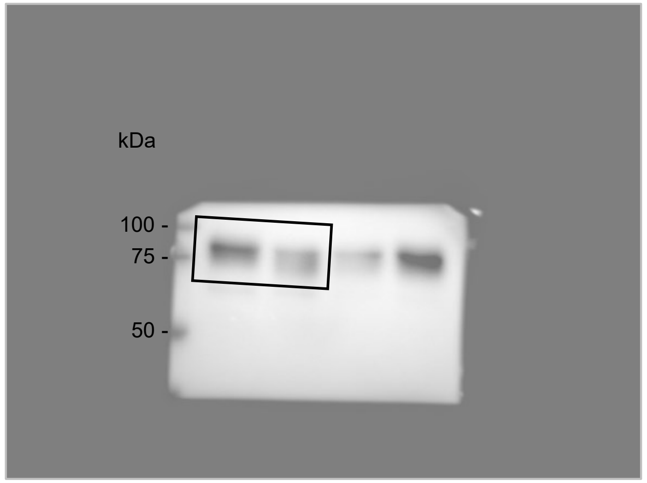

α-Tubulin for FOXJ3

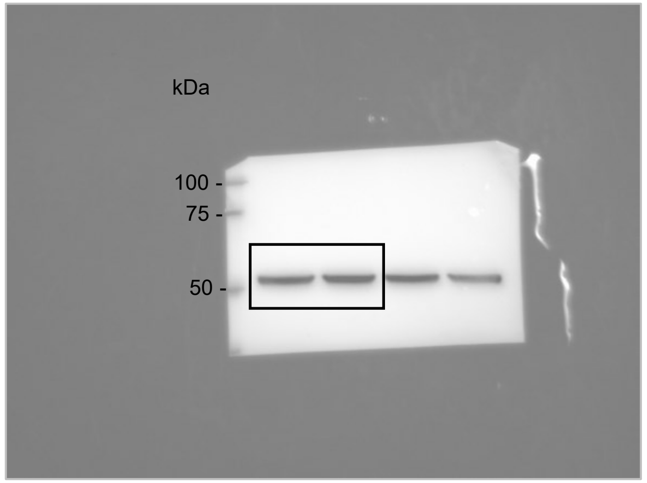

Figure S1E

HCT116 - Scr_KD and miR-27a_KD

FOXJ3

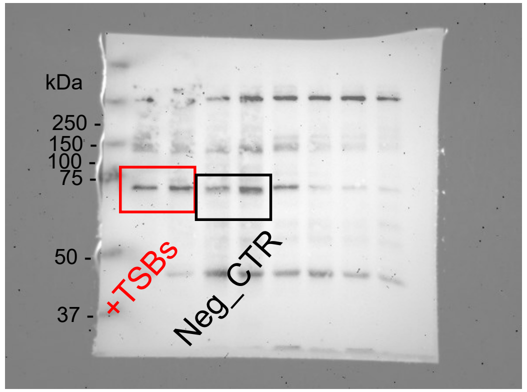

α-Tubulin for FOXJ3

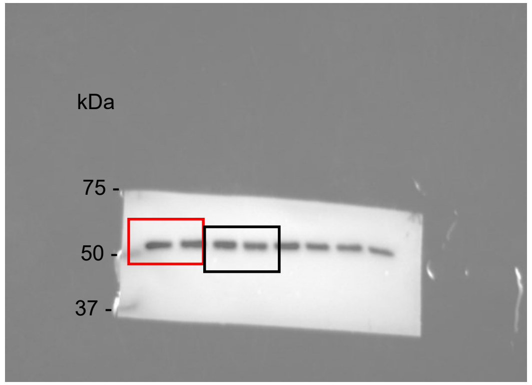

HT29 - Scr_OE and miR-27a_OE

FOXJ3

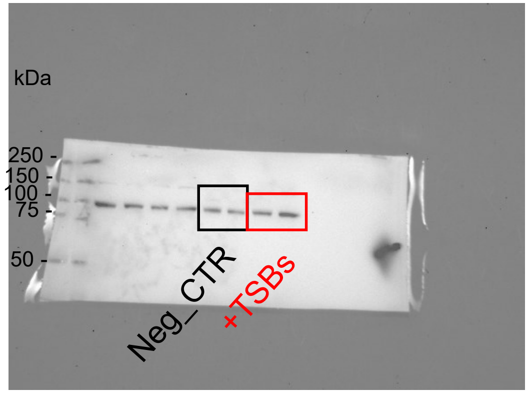

α-Tubulin for FOXJ3

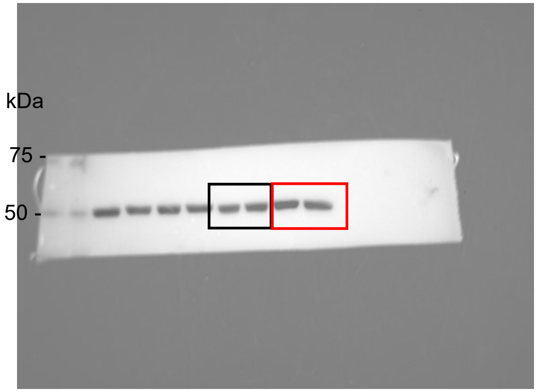

Figure S2

Panel **A** (HCT116 - Scr_KD and miR-27a_KD)

PGC1-α

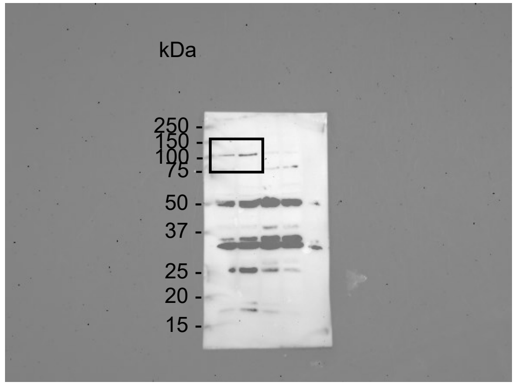

NRF1

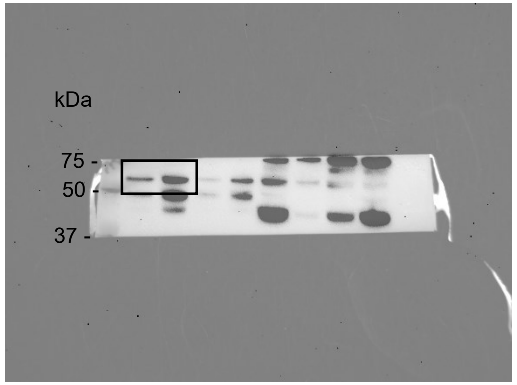

TFAM

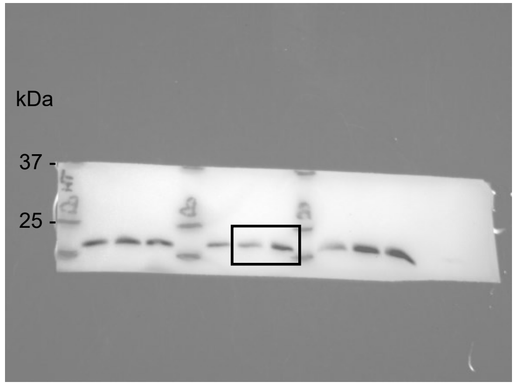

α-Tubulin for PGC1-α (not shown)

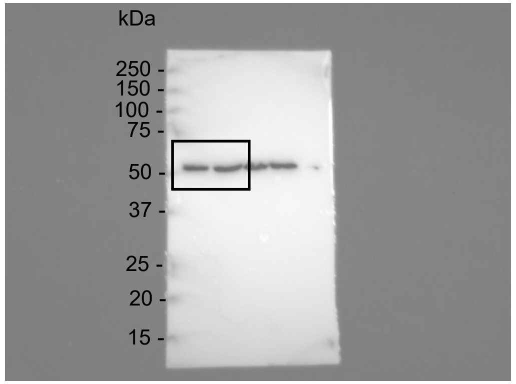

α-Tubulin for NRF1

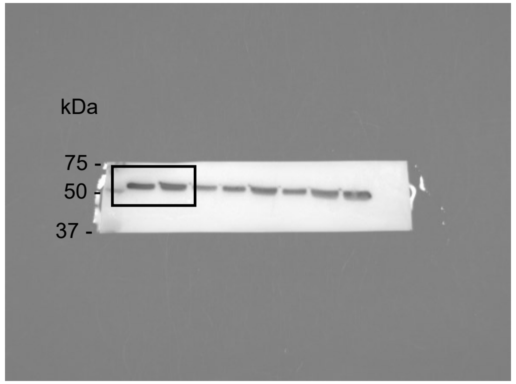

α-Tubulin for TFAM (not shown)

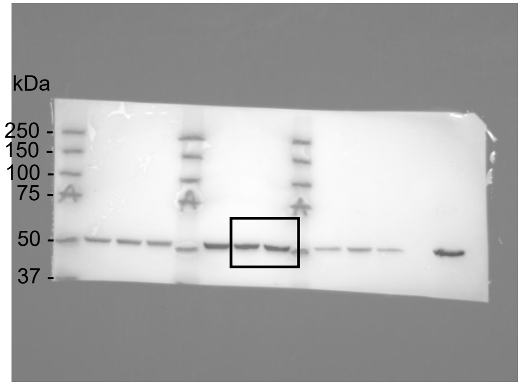

Figure S2

Panel **A** (HT29 - Scr_OE and miR-27a_OE)

PGC-1α

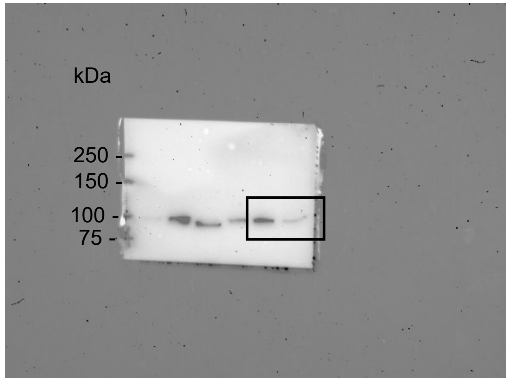

NRF1

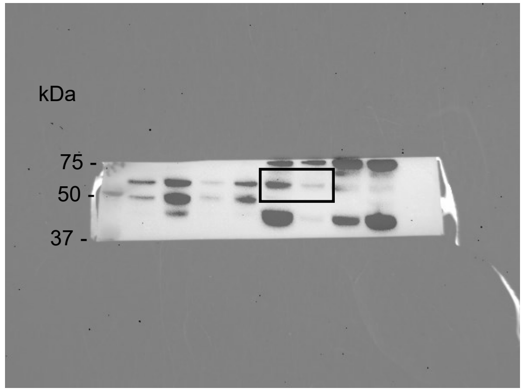

TFAM

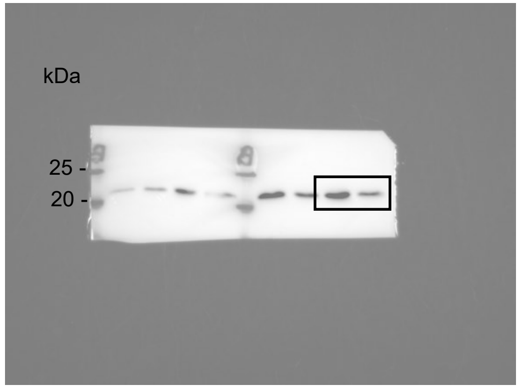

α -Tubulin for PGC1- α (not shown)

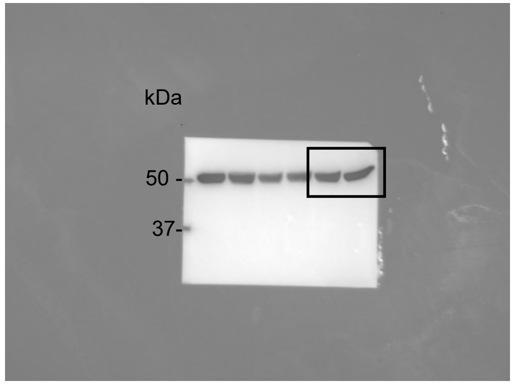

α -Tubulin for NRF1 (not shown)

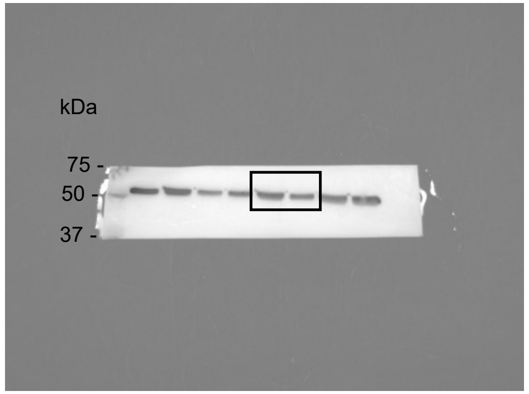

α -Tubulin for TFAM

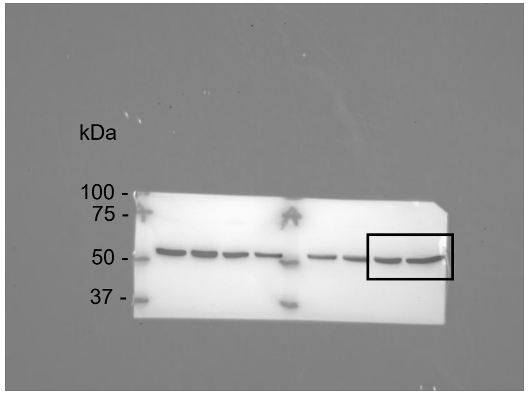

Figure S2

Panel **C** (HCT116 - Scr_KD and miR-27a_KD)

PGC1-α

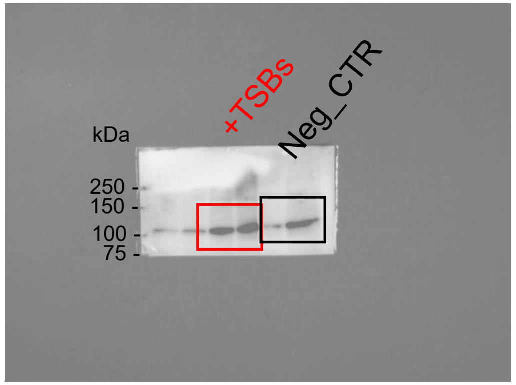

NRF1

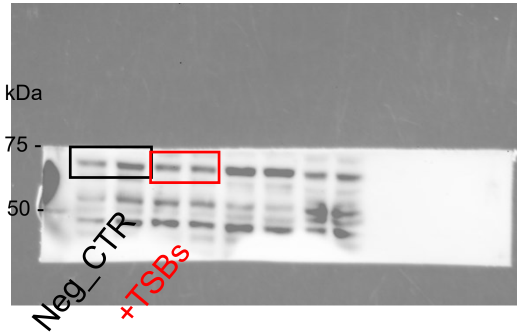

TFAM

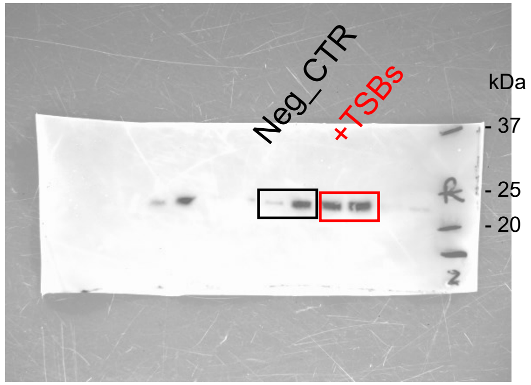

α -Tubulin for PGC1- α (not shown)

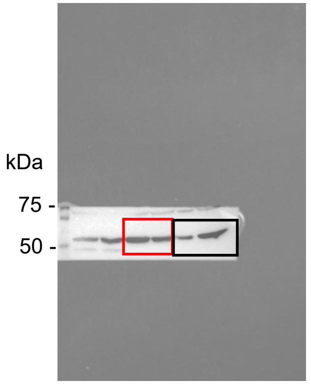

α -Tubulin for NRF1

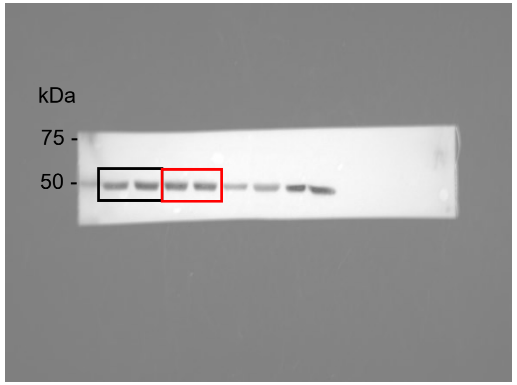

α -Tubulin for TFAM (not shown)

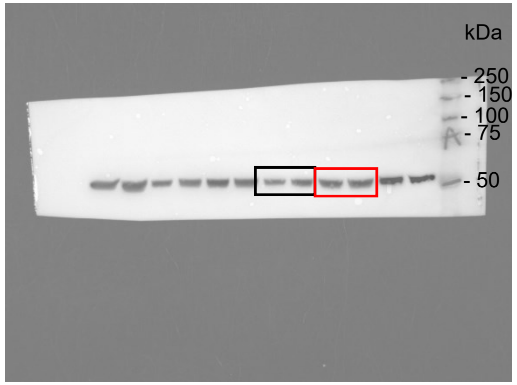

Figure S2

Panel **C** (HT29 - Scr_OE and miR-27a_OE)

PGC-1α

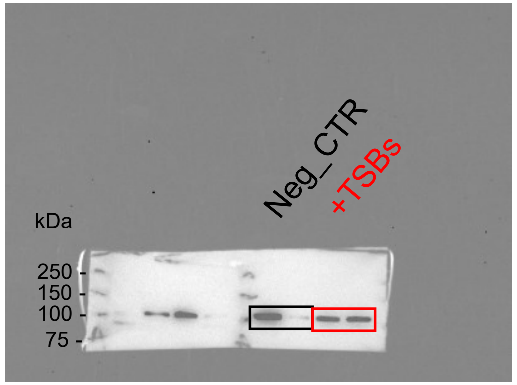

NRF1

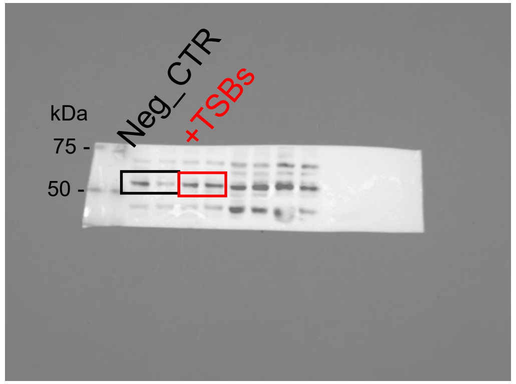

TFAM

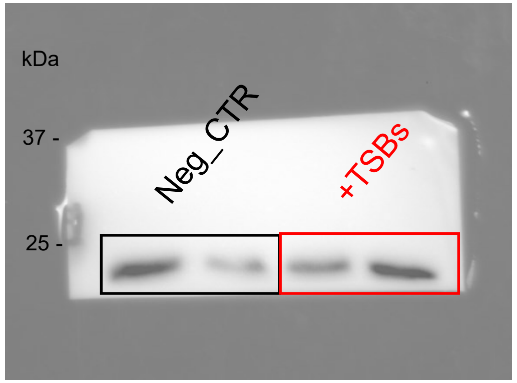

α-Tubulin for PGC-1 α (not shown)

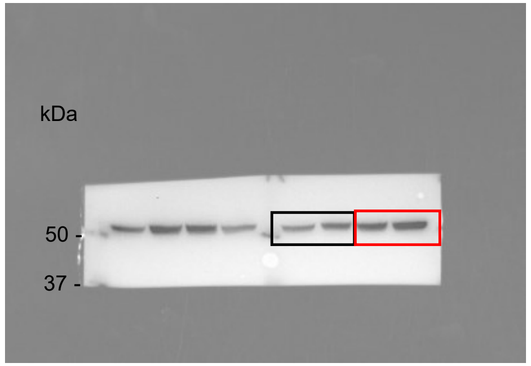

α-Tubulin for NRF1

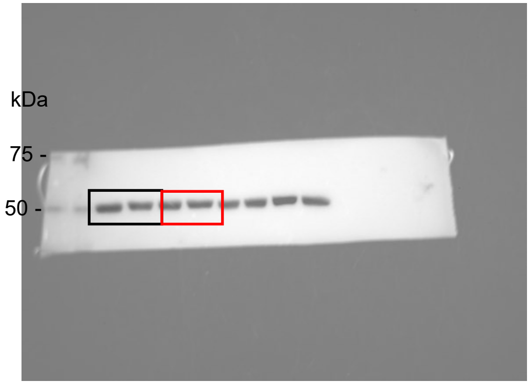

α-Tubulin for TFAM (not shown)

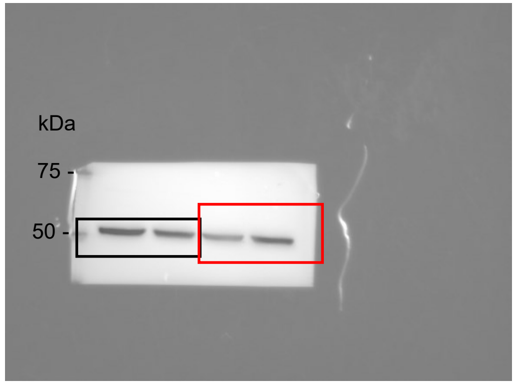

Figure S3
Panel **B**

HCT116 - Scr_KD and miR-27a_KD

OXPHOS

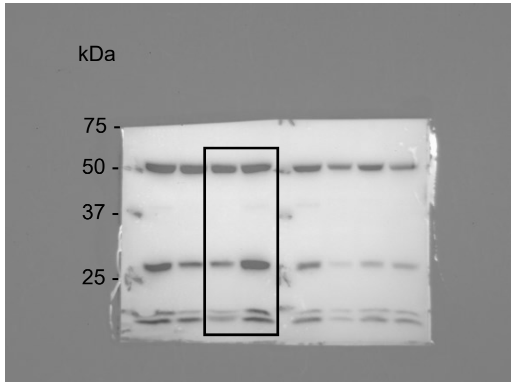

α-Tubulin for OXPHOS

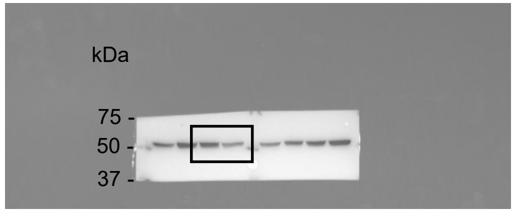

HT29 - Scr_OE and miR-27a_OE

OXPHOS

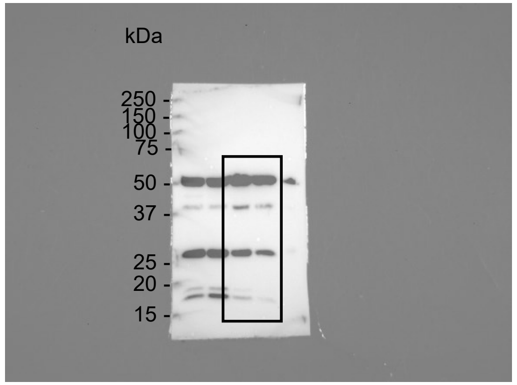

α-Tubulin for OXPHOS

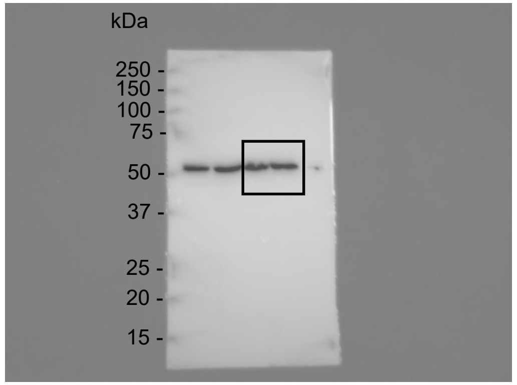

Figure S3
Upper panel **D** (HCT116 - Scr_KD and miR-27a_KD)

MFN1

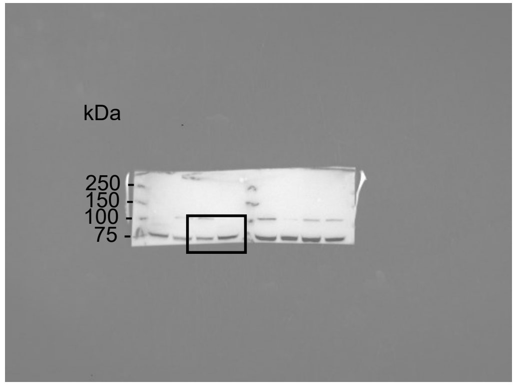

MFN2

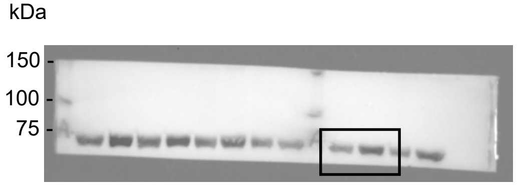

OPA1

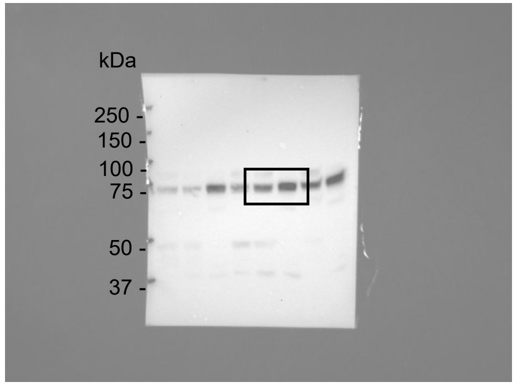

α-Tubulin for MFN1 (not shown)

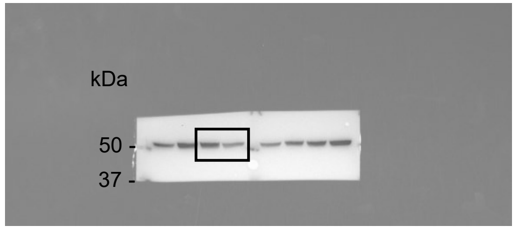

α-Tubulin for MFN2

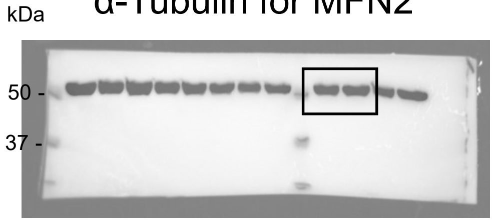

α-Tubulin for OPA1 (not shown)

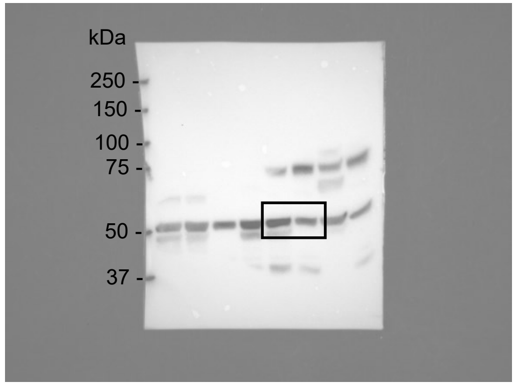

Figure S3
Lower panel D
(HCT116 - Scr_KD and miR -27a_KD)

MFF

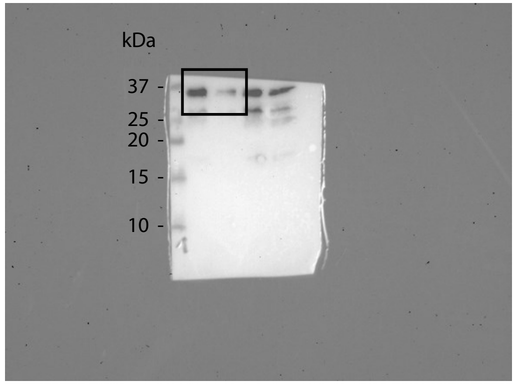

α-Tubulin for MFF (not shown)

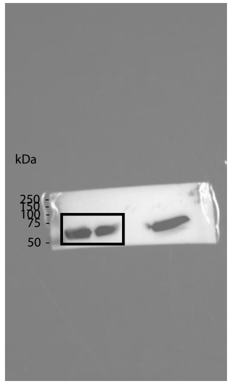

p-DRP1 (S616)

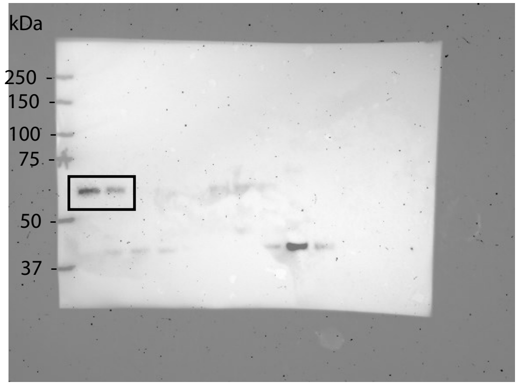

DRP1

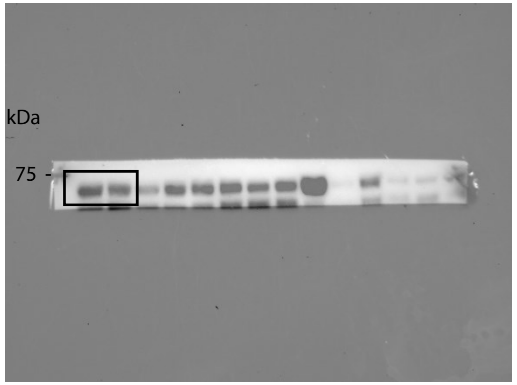

α-Tubulin for p-DRP1 (S616) and DRP1 (not shown)

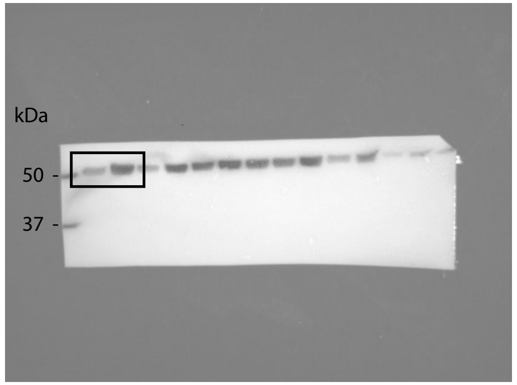

p-DRP1 (S637)

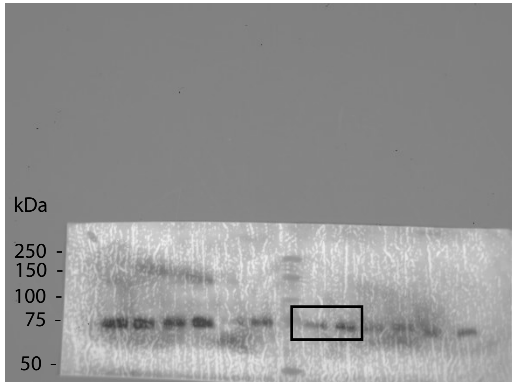

DRP1 (not shown)

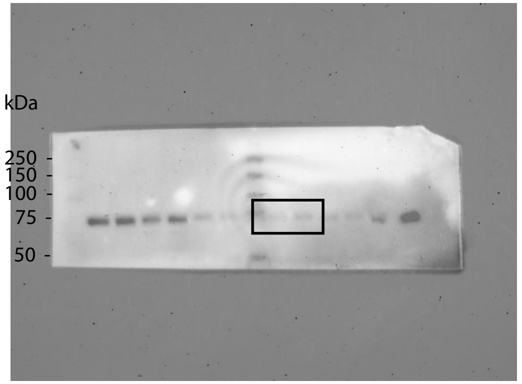

α-Tubulin for p-DRP1 (S637) and DRP1

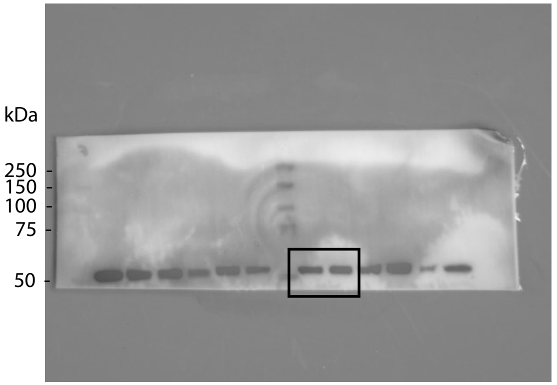

Figure S3

Upper panel **D** (HT29 - Scr_OE and miR-27a_OE)

MFN1

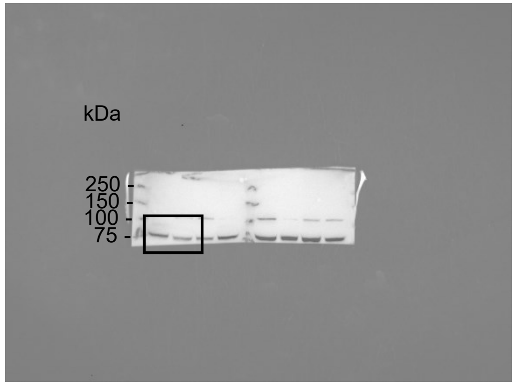

MFN2

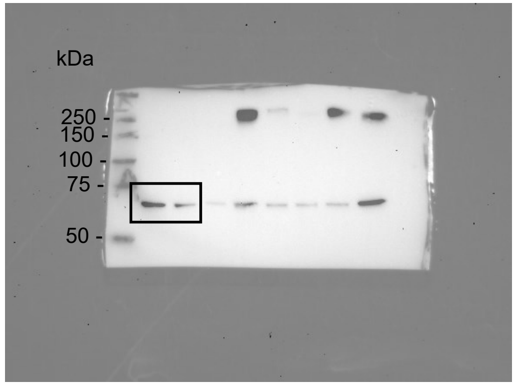

OPA1

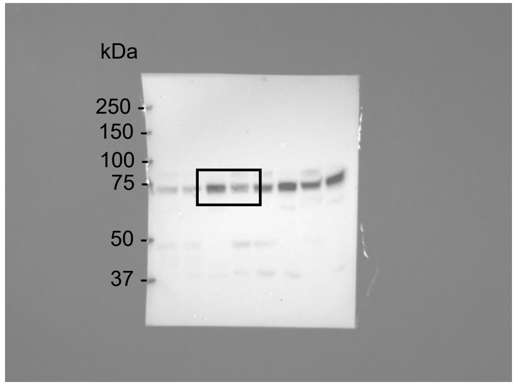

α-Tubulin for MFN1 (not shown)

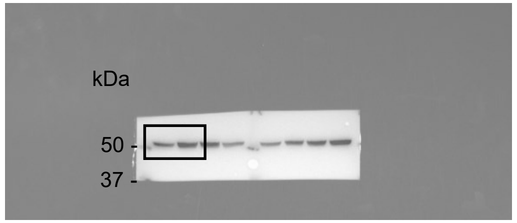

α-Tubulin for MFN2

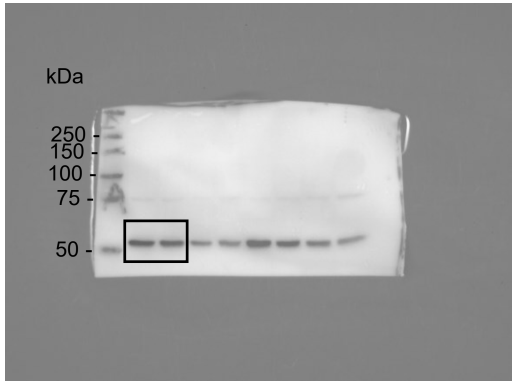

α-Tubulin for OPA1 (not shown)

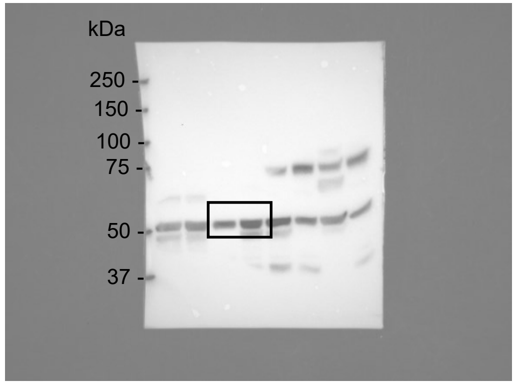

Figure S3
Lower panel **D**
(HT29 - Scr_OE and miR-27a_OE)

MFF

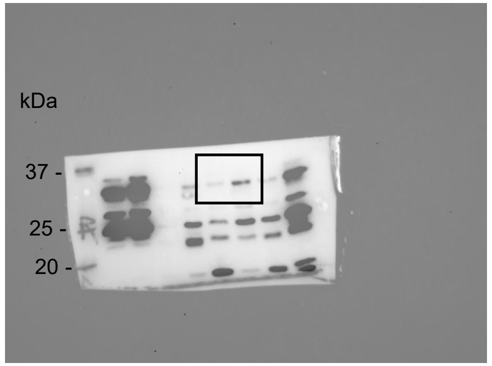

α-Tubulin for MFF

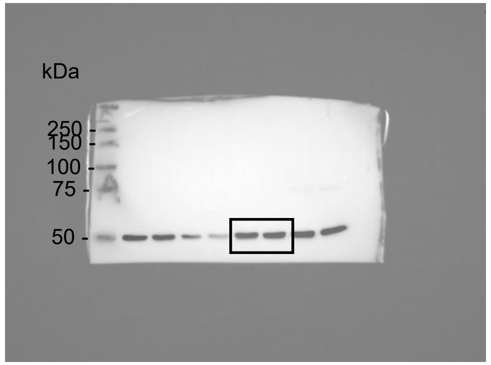

p-DRP1 (S616)

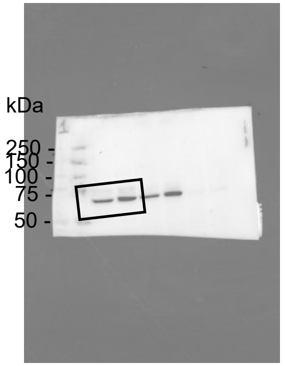

DRP1

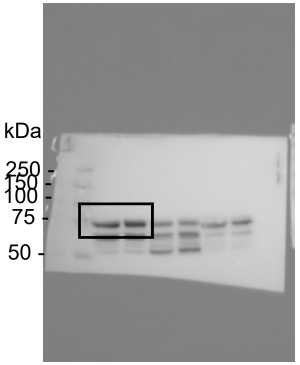

α-Tubulin for p-DRP1 (S616) and DRP1 (not shown)

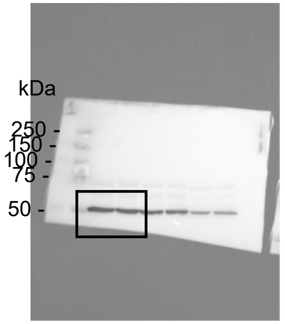

p-DRP1 (S637) flipped

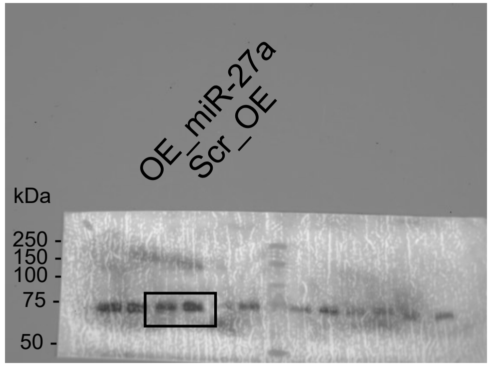

DRP1 flipped

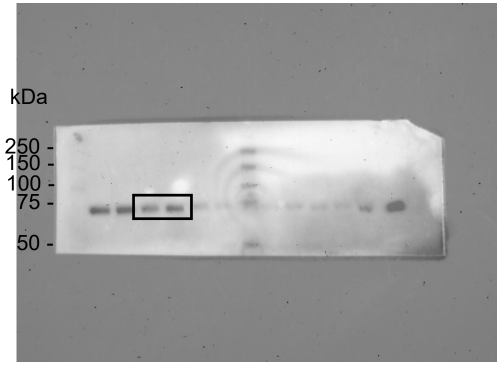

α-Tubulin for p-DRP1 (S637) and DRP1 flipped (not shown)

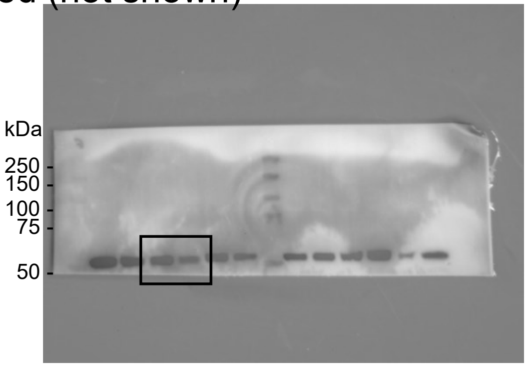

Figure 6 A
(HCT116)

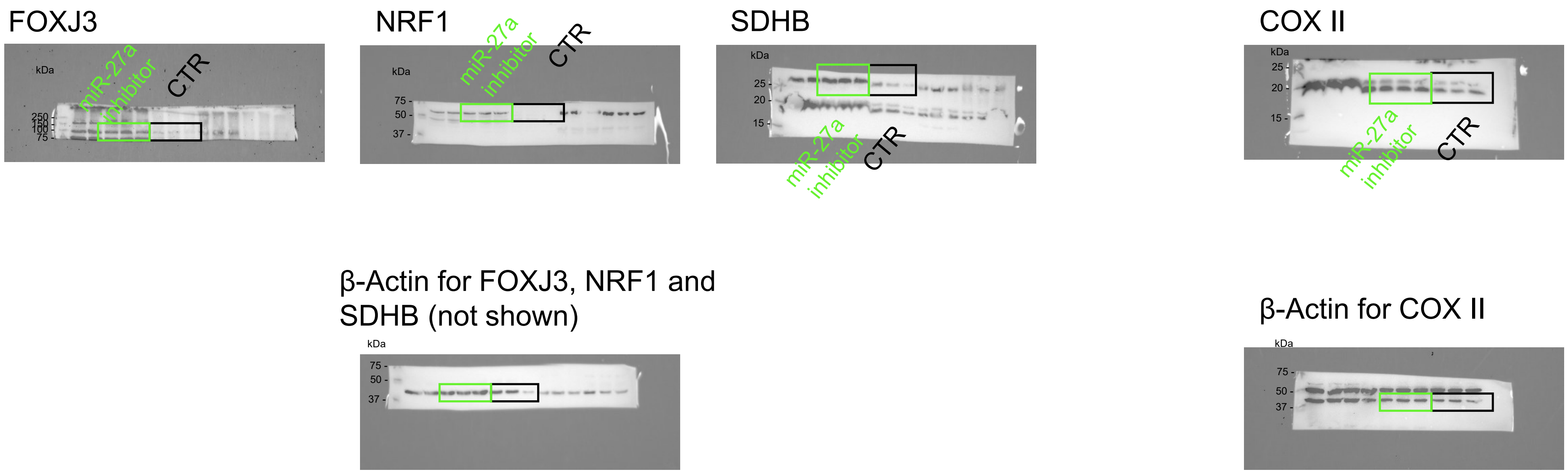

(HT29)

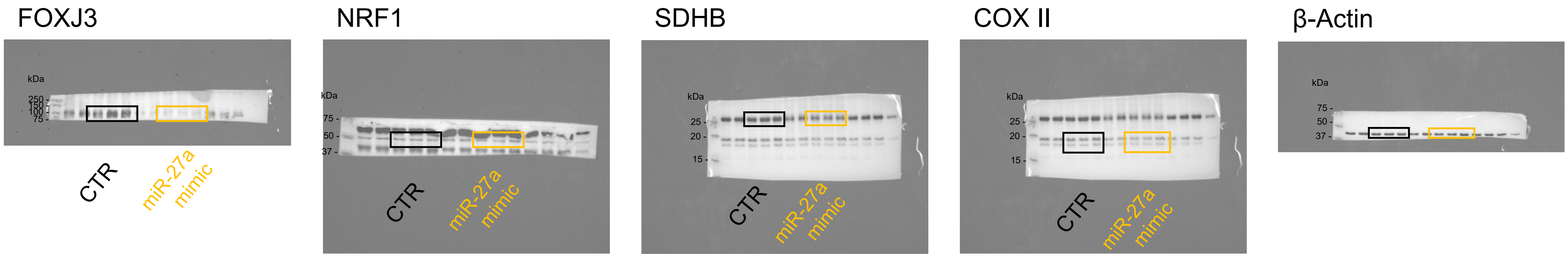

Supplementary Figure S1B

HCT116

FOXJ3

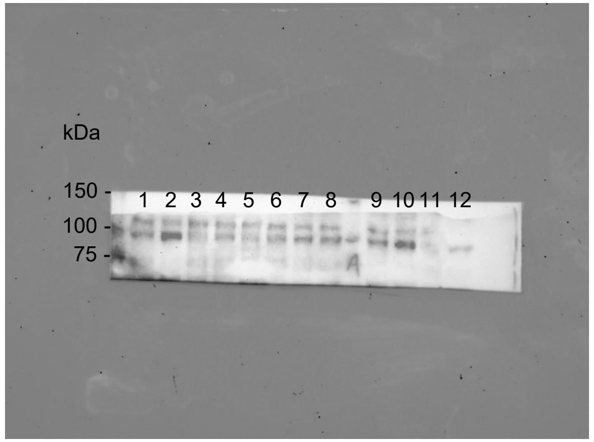

Order shown in the figure:
9: Scr_KD
10: mir-27a_KD
1: Scr_KD + Neg_CTR
2: mir-27a_KD + Neg_CTR
3: Scr_KD + TSB1
4: mir-27a_KD + TSB1
5: Scr_KD + TSB2
6: mir-27a_KD + TSB2
7: Scr_KD + TSBs
8: mir-27a_KD + TSBs
11 and 12: not shown

α-Tubulin for FOXJ3

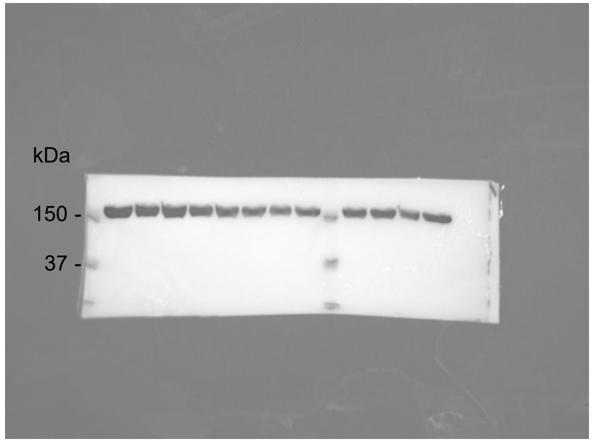

HT29

FOXJ3

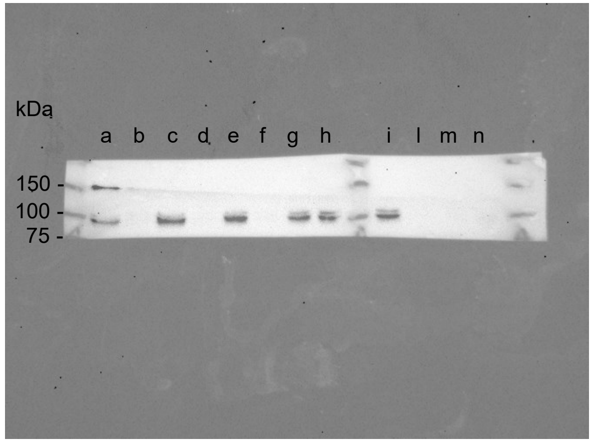

Order shown in the figure:
i: Scr_OE
l: mir-27a_OE
a: Scr_OE + Neg_CTR
b: mir-27a_OE + Neg_CTR
c: Scr_OE + TSB1
d: mir-27a_OE + TSB1
e: Scr_OE + TSB2
f: mir-27a_OE + TSB2
g: Scr_OE + TSBs
h: mir-27a_OE + TSBs
m and n: not shown

α-Tubulin for FOXJ3

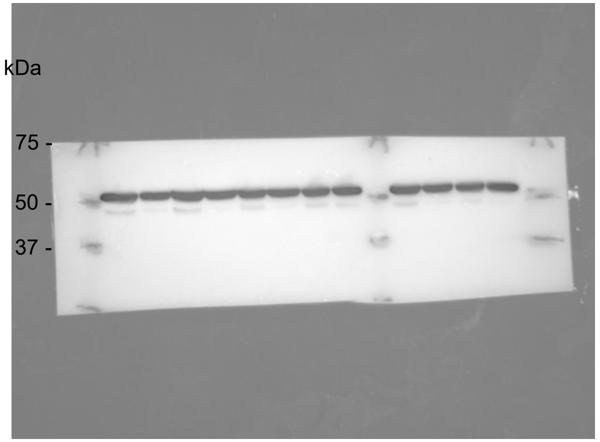

Supplement: Supplementary file 1 [file cancers-13-04994-s001.zip › cancers-1405776-supplementary.pdf]
